# More closely related plants have more distinct mycorrhizal communities

**DOI:** 10.1093/aobpla/plu051

**Published:** 2014-09-23

**Authors:** Kurt O Reinhart, Brian L Anacker

**Keywords:** Functional complementarity, host identity, mixed-grass prairie, mycorrhizal community structure, niche partitioning, phylogenetic host specificity, phylogenetic signal, plant–soil (below-ground) interactions.

## Abstract

Neighbouring plants are known to vary from having similar to dissimilar arbuscular mycorrhizal fungal (AMF) communities. One possibility is that closely related plants have more similar AMF communities than more distantly related plants, an indication of phylogenetic host specificity. Here, we investigated the structure of AMF communities among dominant grassland plants at three sites in the Northern Great Plains to test whether the pairwise phylogenetic distance among plant species was correlated with pairwise AMF community dissimilarity. For eight dominant and co-occurring grassland plant species, we reconstructed a phylogeny based on DNA data and characterized the AMF communities of their roots at each site. Community analyses revealed that AMF communities varied among sites and among plant species. Contrary to expectations for phylogenetic host specificity, we found that within a site more closely related plants had more distinct AMF communities despite their having similar phenologies. Associations with unique AMF communities may enhance the functional complementarity of related species and promote their coexistence.

## Teaser

We observed that mycorrhizal communities were more divergent among closely related plant species than among distantly related plant species. This was counter to the observation that plant mutualists (e.g. pollinators, seed dispersers) are often shared among closely related host plant species. Since mycorrhizae may affect nutrient competition among neighboring plants, closely related plant neighbors that associate with unique mycorrhizae may have greater functional complementarity and a greater capacity to coexist.

## Introduction

Ninety percent of plants are estimated to associate with arbuscular mycorrhizal fungi (AMF) (Wang and Qiu 2006). Neighbouring pairs of plants can associate with similar or distinct AMF (e.g. Vandenkoornhuyse *et al.* 2003; Stukenbrock and Rosendahl 2005; Montesinos-Navarro *et al.* 2012*b*; Osanai *et al.* 2013). A persistent challenge is to understand the source(s) of this variability and the interactions between plants and soil biota. Such knowledge may yield mechanistic insights into how AMF contribute to plant coexistence, plant diversity and plant productivity (e.g. Hart *et al.* 2003; Hausmann and Hawkes 2009; Davison *et al.* 2011; Montesinos-Navarro *et al.* 2012*a*; Morris *et al.* 2013).

Mutualist and antagonist host associations have been shown to exhibit phylogenetic signal (Kawakita *et al.* 2010; Poulin *et al.* 2011), which we define as the tendency for close relatives to resemble each other more closely in their characteristics than expected by chance (Wiens *et al.* 2010). For example, mycorrhizae (Jacquemyn *et al.* 2011), seed dispersers (Rezende *et al.* 2007*a*), plant pathogens (e.g. Gilbert and Webb 2007) and insect herbivores (e.g. Weiblen *et al.* 2006) are all known to associate with closely related host species. Phylogenetic conservatism in host use is also called ‘phylogenetic host specificity’ (e.g. Veresoglou and Rillig 2014). Understanding the causes of phylogenetic signal for even a single trait is difficult, as numerous factors can limit changes over evolutionary time (e.g. slow diversification rates, insufficient time, strong selection, morphological constraints and trade-offs; Wiens *et al.* 2010). Phylogenetic signal in mutualist and antagonist host interactions is even more complex, as multiple traits often mediate species interactions, selection on interactions can be indirect or highly variable in time, and each partner may reciprocally influence the other (e.g. coevolution between host and partner).

Plant associations with AMF and/or responses to AMF have been shown to exhibit phylogenetic signal, at both broad phylogenetic scales (Wang and Qiu 2006) and among local plant assemblages (Reinhart *et al.* 2012). A potential ecological explanation for the phylogenetic signal for AMF associations among co-occurring plants is phenological filtering (convergence of AMF communities among plants in response to their having similar phenologies). Phenological filtering may contribute to phylogenetic host specificity because (i) closely related plants often share phenological schedules (e.g. Davies *et al.* 2013) and (ii) AMF taxa are highly seasonal (e.g. Dumbrell *et al.* 2011). Phenological synchrony among plants and AMF would mean that plants with distinct phenologies are likely to associate with a different pool of AMF than plants with overlapping phenologies. This might then result in a positive correlation between the host phylogenetic distance and the distance in AMF associations. A perhaps less-likely explanation is that closely related plants may be interconnected by common hyphal networks that improve the establishment of relatives' seedlings (Van Der Heijden and Horton 2009). Related plants may also associate with ‘more’ divergent AMF communities than expected by chance, based on one study (Veresoglou and Rillig 2014). Their study provided several interpretations for this including that closely related plants partition the AMF pool in ways that promote plant functional complementarity. For example, neighbouring grasses that share many traits may have increased their functional complementarity by associating with unique AMF. The well-known positive correlation of AMF diversity and plant productivity indicates a role of AMF in functional complementarity (Van Der Heijden *et al.* 1998*b*). The direction of the phylogenetic signal, then, may depend in part upon the hierarchical balance of abiotic and biotic filters.

Here our goal was to characterize the AMF taxa associated with eight grassland plant species within three sites in the Northern Great Plains of North America. We also tested whether related plant species have similar phenologies and AMF. In addition to testing for a phylogenetic signal in plant phenology, we tested whether pairwise phylogenetic distance among plants predicts the similarity in the AMF found in their roots in the field. Contrary to the expectation of phylogenetic host specificity, we found that phylogenetic distance and AMF distance were negatively correlated, indicating phylogenetic divergence in host use (e.g. close plant relatives hosted different AMF) and a possible role for below-ground plant–AMF associations in niche partitioning and coexistence of confamilial grasses.

## Methods

### Study species, sites and system

We used plant community composition data for the three sites from a previously published study on host-specific soil biota effects (also called soil feedback effects) (Reinhart 2012) to select the eight most frequent and abundant plants across all sites. Our focus on dominant species limited our sample size relative to all possible species occurring across sites but ensured pairs of species typically co-occurred in nature and did not occupy separate portions of an undescribed local gradient (Table 1). The sample included one perennial sedge (*Carex filifolia*), one exotic annual C3 grass (*Bromus japonicus*), three perennial C3 grasses (*Hesperostipa comata*, *Koeleria macrantha*, *Pascopyrum smithii*), one perennial C4 grass (*Bouteloua gracilis*), one exotic biennial forb (*Tragopogon dubius*) and one perennial subshrub (*Artemisia frigida*). In other words, the eight dominant plant species included five from Poaceae, one from Cyperaceae and two from Asteraceae. This distribution resulted in 35 % of our pairwise comparisons being intra-family comparisons of Poaceae taxa. A related study included relatively few studies (we determined 3 of 10 grassland studies) with two or more Poaceae taxa (Veresoglou and Rillig 2014). That said, our comparisons do not include very close or closest (i.e. sister) relatives. A review of the 342 plant species known to occur at the Fort Keogh Livestock & Range Research Laboratory (22 000 ha) indicates sister taxa exist at the site for half the species (i.e. *Artemisia*, *Bromus*, *Bouteloua* and *Carex*). 

**Table 1.** Eight most dominant plant species across three sites with descriptions of plant abundance and AMF communities in roots of focal species. *Plant relative abundance data were from 2008 (Reinhart 2012).

Sites	Plant species	Relative abundance in the field (mean % cover, % of 1 m^2^ plots with species)*	Percentage of root samples with AMF	AMF OTU richness
1	*Artemisia frigida*	2.3, 90	50	1
	*Bouteloua gracilis*	0.5, 100	50	4
	*Bromus japonicus*	10.7, 100	25	5
	*Carex filifolia*	12.1, 100	25	1
	*Hesperostipa comata*	58.1, 100	62.5	5
	*Koeleria macrantha*	2.4, 70	50	2
	*Pascopyrum smithii*	0.4, 100	37.5	2
	*Tragopogon dubius*	4.9, 100	12.5	1
2	*Artemisia frigida*	1.5, 10	75	2
	*Bouteloua gracilis*	0.8, 80	50	5
	*Bromus japonicus*	3.1, 100	75	5
	*Carex filifolia*	12.3, 40	37.5	2
	*Hesperostipa comata*	52.5, 100	37.5	3
	*Koeleria macrantha*	1.7, 80	50	7
	*Pascopyrum smithii*	6.7, 90	25	2
	*Tragopogon dubius*	13.1, 100	50	3
3	*Artemisia frigida*	9.3, 90	0	0
	*Bouteloua gracilis*	0.1, 60	25	7
	*Bromus japonicus*	3.7, 90	50	1
	*Carex filifolia*	32.6, 100	50	2
	*Hesperostipa comata*	31.8, 100	62.5	6
	*Koeleria macrantha*	3.2, 100	0	0
	*Pascopyrum smithii*	4.9, 100	37.5	3
	*Tragopogon dubius*	0.9, 80	0	0

Most related studies that characterized AMF of more than one plant species (reviewed by Veresoglou and Rillig 2014) sampled only a single site. We sampled three replicate sites to avoid site-specific patterns. Sites were separated by 9–77 km in Custer County, Montana, USA, consisted of loamy soils and represented one of the dominant grassland types in the region (*H. comata*, *B. gracilis* and *C. filifolia*) (Martin *et al.* 1998). The sites were located in the Northern Great Plains Steppe ecoregion of North America which is dominated by semiarid mixed-grass prairie that cover >22 million hectares (Martin *et al.* 1998). Peak annual productivity for this system occurs between June and July reflecting its dominance by C3 graminoids and the grasslands are primarily nitrogen and not phosphorus limited (K.O.R., unpubl. data). Further details on the study sites (Reinhart 2012) and system (Martin *et al.* 1998) are reported elsewhere.

### Plant phylogeny construction

We reconstructed a phylogeny for eight angiosperm species based on DNA data and the Bayesian analysis. We searched the GenBank database for sequences of five loci often used in published phylogenies: *ITS1*, *matK*, *rbcL*, *trnL* and *5.8s* (e.g. Reinhart *et al.* 2012). Multiple loci are necessary to differentiate plant species because some loci represent conservative coding regions (e.g. *rbcL*) and others represent more rapidly evolving portions (e.g. *matK*) (e.g. Reinhart *et al.* 2012). When a sequence was not available for the exact same species for a marker, we used sequences available for species of the same genus, and also native to North America (acquired *rbcL* sequences for two species). All species had at least three loci represented for it or a congener in GenBank (http://www.ncbi.nlm.nih.gov). We included an ancestral angiosperm species (*Magnolia grandiflora*) as an outgroup to root the molecular phylogeny. We followed a previously described workflow to align sequences, edit sequences and determine the best-fit maximum likelihood models of nucleotide substitution (*sensu*Reinhart *et al.* 2012). Using the concatenated sequence alignments, we performed a partitioned Bayesian inference, estimating the posterior probability distribution of all possible phylogenies using a Markov chain Monte Carlo algorithm (i.e. Metropolis algorithm) implemented in MrBayes version 3.1.2 (Huelsenbeck and Ronquist 2001). Two independent Markov chains were run, each with four heated chains for 500 000 generations. The final average standard deviation of split frequencies was 0.0002, indicating good convergence of the two independent runs. We sampled runs every 50 generations and used a burnin of 500 trees to generate a majority rule consensus tree (i.e. phylogram). The resulting phylogram was transformed with non-parametric rate smoothing into an ultrametric tree using APE version 2.5 (Paradis *et al.* 2004) in R (R Developement Core Team 2011). The ultrametric tree, which has unit-less branch lengths, is shown in Fig. 2. We also ran divergence time estimation to get branch lengths in millions of years based on several calibration dates; we noted that measures of phylogenetic signal for phenology and AMF distance were qualitatively similar - see supporting information.

### Root collection

We sampled eight mature plants per species and per site for a total of 192 samples (8 species × 8 plants × 3 sites). The plants were randomly sampled from each site in a 50 × 20-m plot. We did not measure the location of individual plants and are not able to address whether AMF community similarity exhibits spatial autocorrelation. We sampled at anthesis because AMF were most likely to be associated with roots when plants were actively photosynthesizing. Detailed description and justification of root sampling are provided in the supporting information.

### AMF communities

We characterized the AMF community in each sampled plant using DNA-based methods involving the construction of a terminal restriction fragment length polymorphism (T-RFLP) library. A main aim of many microbial community ecology studies is to quantify microbial species richness and attach the best available names to their operational taxonomic units. Methods with a more limited capacity to differentiate and name microbial species (e.g. T-RFLP, phospholipid-derived fatty acid profiles) may still be used to quantify differences in microbial community composition. For example, a recent study determined that differences in fungal community composition among treatments were comparable whether based on data derived from T-RFLP or pyrosequencing despite the latter's detection of many more ‘species’ (e.g. Dhami *et al.* 2013).

Sample processing, DNA extraction, amplification with nested polymerase chain reaction (PCR) and database T-RFLP characterization of AMF communities followed the methods of three previous studies (Renker *et al.* 2003; Aldrich-Wolfe 2007; Jordan *et al.* 2012). Detailed molecular protocols and analysis procedures are provided in supporting information. In brief, three root fragments (6 mm length) per sample were combined and used to extract DNA. The ITS region was amplified by nested PCR. PCR1 used the primer pair SSU-Glom1 and LSU-Glom1. LSU-Glom1 is specific to the Glomeromycota but also amplifies some basal Basidiomycetes (Renker *et al.* 2003). To further reduce the amplification of DNA from non-AMF, PCR1 amplicons were digested at 37 °C with AluI (Renker *et al.* 2003) see supporting information. The ITS region of most AMF lacks an AluI cut site, while the ITS regions of other fungi likely to have been amplified during PCR1 typically contain multiple AluI cut sites (Renker *et al.* 2003; Aldrich-Wolfe 2007; Jordan *et al.* 2012). The AluI digested products were then amplified for PCR2 with the fluorescently labelled universal fungal primers 6-FAM-ITS4 and HEX-ITS5.

Amplicons from PCR2 were then restriction digested with HinfI and MboI for T-RFLP. Restriction digested amplicons were submitted along with undigested amplicons to a DNA sequencing service centre. Lengths of terminal restriction fragments and undigested amplicons were examined with Peak Scanner Software, version 1 (Applied Biosystems, Foster City, California, USA).

The package TRAMPR implemented in the R environment was used to match PCR2 amplicons and terminal restriction fragments (TRFs) from root samples with a database of AMF knowns and to provide output for additional analyses (FitzJohn and Dickie 2007). A database of known AMF was built by aggregating two existing databases including one with isolates from around the world (Appendix C in Aldrich-Wolfe 2007) and another that expanded this original database by including isolates specific to the Northern Great Plains steppe ecoregion (Jordan *et al.* 2012). The two prior studies also characterized AMF communities with the same primers and T-RFLP. We supplemented the database further with additional isolates of local AMF as done by these prior studies. TRAMPR was used to accurately detect AMF by matching sets of TRFs and PCR2 amplicons from our samples with sets from our AMF knowns database. This was a conservative approach that helped reduce the probability that TRFs of non-AMF, persisting after AluI restriction enzyme digests, produced false-positive counts of AMF that were not actually present. Detailed protocols are provided see supporting information. TRAMPR was then used to generate a presence/absence matrix for each of the 192 samples. Hereafter, each known cluster profile is referred to as an operational taxonomic unit (OTU).

### The distribution of AMF by site and species

Similarity of AMF communities within vs. among plant species and grassland sites was tested using the non-parametric permutation test ADONIS, with 1000 permutations, in the ‘vegan’ package in R (Oksanen *et al.* 2011). For Site 1, one plant species (*T. dubius*) was removed from the dataset because we detected AMF for only one of eight samples. For Site 3, three species (*A. frigida*, *K. macrantha* and *T. dubius*) were omitted because no AMF OTUs were detected. To visualize the (dis)similarities in AMF communities among plant species and sites, we performed a non-metric multidimensional scaling (NMDS) ordination using the function ‘metaMDS’ in vegan which converged after three restarts. Prior to performing the NMDS, we summed the sample data per species (per site) and standardized the data with ‘decostand’ function with method = ‘total’ in vegan. ANOVA was used to model the effect of plant species and grassland site on OTU richness using Proc GLM in SAS version 9.3 (SAS Institute, Inc., Cary, NC, USA). Plant species and site were fixed effects. Prior to running the ANOVA, the treatment group variances were determined to be homogeneous.

### Phylogenetic signal tests

We separately tested whether plant phenology and AMF associations varied by plant phylogeny and exhibited a phylogenetic signal. To assess the degree phylogeny explains the phenological similarity of species (i.e. trait conservatism), we quantified the phylogenetic signal using the descriptive *K* statistic using the package Picante (Kembel *et al.* 2010) in R. The *K* statistic compares the observed signal for a plant trait (i.e. mean Julian day of anthesis across sites) with the signal under a Brownian motion process and provides an estimate of phylogenetic signal or trait conservatism. *K* values <1 indicate that species resemble each other less than expected under Brownian motion evolution; *K* values >1 indicate that species resemble each other more than expected. Picante uses a permutation test to test the significance of the observed *K* value. To do this, the names of taxa are iteratively shuffled across the tips of the phylogeny 999 times. The significance of the *K* statistic is assessed with the quantile of the observed phylogenetically independent contrast variance vs. the null distribution, which provides a one-tailed *P*-value testing whether the phylogenetic signal is greater than expected. With only eight species, the significance test should be interpreted cautiously since tests with very few species are likely to fail to detect significant differences when they actually exist (Type II errors) (Rezende *et al.* 2007*b*) and justified use of a relaxed alpha level of 0.10.

To test for a phylogenetic signal in AMF host associations (i.e. phylogenetic host specificity), we determined whether variation in AMF distance (community dissimilarity) among pairs of host plant species was positively related to the phylogenetic (ultrametric) distance between this pair of plant hosts. Phylogenetic distance between each pair of plant species was determined with the program Patristic. Arbuscular mycorrhizal fungal distance among pairs of plant species per site was measured using Jaccard distance implemented with the package vegan (Oksanen *et al.* 2011) in R. Prior to Jaccard distance calculations, we summed the sample data (i.e. binomial presence/absence data) by species per site. This produced quantitative data of AMF OTU associations per plant species per site. We treated sites as a form of landscape-level replication and averaged dissimilarity measures for each pair of species (e.g. *A. frigida* and *C. filifolia*) which reduced the data to 28 species pairs (for a pool of 8 species there are 28 species pairs). This approach ensures that our pairwise description of pairwise AMF distance is not site specific. We used linear regression (LR) to determine the direction of the relationship between AMF distance and phylogenetic distance. A Mantel test was used to account for the non-independence in the data (not accounted for with LR), caused by a given plant species being present in multiple pairwise species combinations (e.g. Weiblen *et al.* 2006; Violle *et al.* 2011). The data were also analysed with quantile regression. Outliers were identified based on the maximum normal residual method (Snedecor and Cochran 1989) and *P* < 0.05. One outlier was detected, removed and the LR and Mantel analyses were repeated. We also tested the sensitivity of the LR results to any one particular species by removing data for each species and repeating LR analyses.

## Results

### The distribution of AMF by site and species

**Figure 1.**
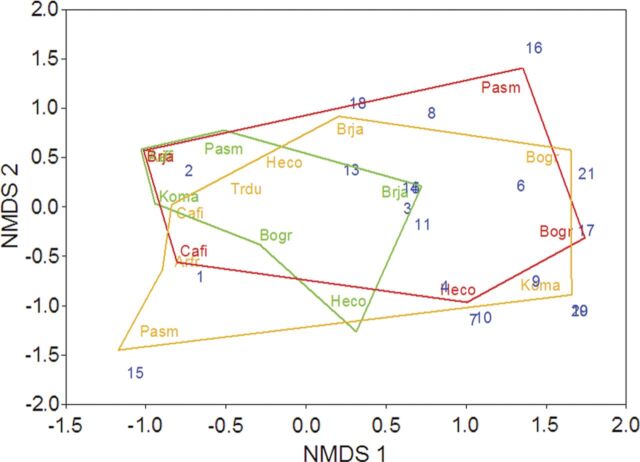
Non-metric multidimensional scaling analysis of AMF community structure among dominant plant species at three grassland sites (Sites: 1 = green, 2 = orange and 3 = red). AMF OTU labels are in blue. Plant species abbreviations are as follows: Arfr, *Artemisia frigida*; Bogr, *Bouteloua gracilis*; Brja, *Bromus japonicus*; Cafi, *Carex filifolia*; Heco, *Hesperostipa comata*; Koma, *Koeleria macrantha*; Pasm, *Pascopyrum smithii*; Trdu, *Tragopogon dubius*. Coloured hulls enclose the plant species per site. The stress computed for this ordination was 0.09.

The AMF of mixed-grass prairie sites varied by plant species (*R*^2^_ADONIS_ = 0.15, *P* = 0.003) and grassland site (*R*^2^_ADONIS_ = 0.03, *P* = 0.026). We also detected an interaction between plant species and site (*R*^2^_ADONIS_ = 0.18, *P* < 0.001) (Fig. 1). Visual inspection of the NMDS plot (Fig. 1) confirmed the ADONIS results and illustrate the variation in AMF communities among plant species, among sites (coloured hulls), and the interaction between plant species and site. Sites had relatively unique AMF: Sites 1 and 2 shared 50 % of their OTUs, 1 and 3 shared 53 % of their OTUs, and 2 and 3 shared 58 % of their OTUs - see supporting information. On the other hand, richness of OTUs per individual root sample did not vary by grassland site (*F*_2,191_ = 1.96, *P* = 0.14) or plant species (ANOVA, *F*_7,191_ = 1.51, *P* = 0.17), and no interaction between plant species and site was detected (*F*_14,191_ = 0.60, *P* = 0.54). 

### Phylogenetic signal tests

**Figure 2.**
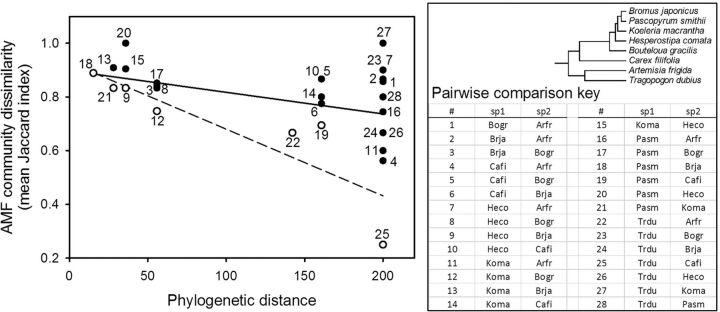
Simple linear regression (solid line) and lower quantile regression (dashed line) for phylogenetic distance among pairs of plant species and mean community dissimilarity of AMF among pairs of host plant species from across three replicate mixed-grass prairie sites. A community dissimilarity value of one would indicate that the AMF communities for two plant species are entirely distinct with no shared associations and the opposite for values of zero. The lower quantile regression was based on the subset of data represented by open circles.

Among the eight plant species, related species tended to have similar phenologies (i.e. anthesis) (phylogenetic signal test, *K* = 0.91, *P* = 0.052) suggesting trait conservatism. The phylogenetic distance of pairs of plants, however, was negatively correlated with AMF distance (*b* = −0.0008, *R*_2_ = 0.15, *F*_1,26_ = 4.67, *P* = 0.040) (Fig. 2) which was also significant according to the Mantel test (Pearson's product–moment correlation, *r* = −0.39, *P* = 0.006) and quantile regression (*P* = 0.003). In other words, related pairs of plants tended to have divergent AMF. Removing each plant species and repeating the regression analyses showed that the slope parameters were consistently negative, but only four of eight models were at least marginally significant (*P* < 0.10). We also detected and removed an outlier comparison (*C. filifolia* and *T. dubius* pair, Jaccard distance value = 0.25) which had relatively minor effects on the LR (*R*^2^ = 0.14, *F*_1,25_ = 4.17, *P* = 0.052), Mantel test (*r* = −0.38, *P* = 0.01) and quantile regression (*P* = 0.0001) (data not shown). As noted in the subsection Phylogenetic signal tests, the Mantel test is the preferred analysis since it accounts for the non-independence in the data. 

## Discussion

### The distribution of AMF by site and species

We characterized the AMF communities of eight dominant plant species co-occurring across three sites of semiarid and temperate grassland of the Northern Great Plains of North America. In other systems, neighbouring grasses were found to have relatively distinct AMF communities (Vandenkoornhuyse *et al.* 2003; Osanai *et al.* 2013). We found that AMF associations among neighbouring plants and species were often distinct in some sites but not in others, reflecting a surprising degree of landscape variation in plant–AMF associations (plant species × site interaction, *P* < 0.001; Fig. 1). As has been shown in pot experiments (Smith and Read 2008), we found limited evidence for host specificity, especially among the more frequently detected AMF OTUs - see supporting information, and the few patterns of host specificity we found were site specific. Among the three comparison grasslands, we found that up to 50 % of AMF OTUs may be site specific.

### Phylogenetic signal tests

We predicted that related plant taxa would share traits (e.g. phenology and AMF associations), an indication of niche conservatism. We envisioned that AMF associations would be affected by a sequential hierarchy of abiotic and biotic filters. First, a temporal filter for co-occurrence of closely related plants and exposure to similar AMF (i.e. phenological filter) (Dumbrell *et al.* 2011; Davies *et al.* 2013). Second, local niche partitioning of AMF by close relatives. We confirmed that more closely related plants tended to have more similar phenologies as has been shown by others (e.g. Davies *et al.* 2013). Contrary to expectations for phylogenetic host specificity, we identified that phylogenetic distance among the dominant plants was negatively correlated with AMF distance (e.g. Fig. 2). Thus, closely related plants tended to have more divergent AMF communities than expected by chance. Another study on plant–AMF associations reported similar findings (Veresoglou and Rillig 2014).

Measures of phylogenetic signal, representative of the entire community, ideally require sampling a large number of species that are broadly distributed across the phylogeny for the community (e.g. Rezende *et al.* 2007*b*). While our study only contained eight species, these were the eight most dominant grassland species (mostly grasses) which helped ensure comparisons were of species that routinely co-occurred (Table 1). The significance of the LRs also depended on the species included in the analyses. However, the slopes of the relationship between phylogenetic distance and AMF distance (community dissimilarity) always remained negative, indicating that the direction of relationship between phylogenetic distance and AMF distance is robust to the influence of any one plant species. Further to the point, we failed to detect any evidence of phylogenetic host specificity (i.e. positive correlation between phylogenetic distance and AMF distance). The grass clade did disproportionately influence our results: the mean pairwise AMF distance among grasses was 0.86 (±0.04 [95 % CI]) vs. 0.75 (±0.08) for grass–non-grass comparisons. Other studies confirm that AMF communities are often relatively distinct among grass species (Vandenkoornhuyse *et al.* 2003; Osanai *et al.* 2013). Unlike our results, a recent meta-analysis reported a marginally significant (*P* < 0.1) positive correlation between plant phylogenetic distance and AMF distance for the subset of data for grasslands (*P* = 0.07) (Veresoglou and Rillig 2014). Their main conclusion, however, was that phylogenetic distance was negatively related to AMF distance. We suspect their atypical finding for the subset of data for grasslands relates to the small number of studies (3 of 10) with more than a single grass species.

Arbuscular mycorrhizal fungal communities are thought to be affected by phenological filtering and temporal fluxes in host availability and photosynthate (e.g. Dumbrell *et al.* 2011). We confirmed that related plants tended to have similar timing of anthesis (e.g. *K* = 0.91, *P* = 0.052) but failed to detect evidence for a positive relationship between phylogenetic distance and AMF distance (e.g. Fig. 2) as done in another study (Veresoglou and Rillig 2014). Many other factors may affect mycorrhizal communities besides phenology and host phylogeny. Mycorrhizal communities may vary among plant species occupying different successional stages or niches (e.g. Davison *et al.* 2011) and vary over sites with varying properties (e.g. soil properties, climate differences, land-use history) (e.g. Hazard *et al.* 2013). Since we sampled plant species that were dominant and co-occurred, it is unlikely that our findings are mainly driven by plants occupying distinct points along local gradients. Other studies using greenhouse experiments have detected soil legacy (or priority) effects where the identity of the plant that initially conditioned or ‘trained’ the soil biota affected the mycorrhizal community of the next plant to establish (e.g. Hausmann and Hawkes 2010; Jordan *et al.* 2012). Natural grassland soils are, however, a diverse mixture of the roots of many species (Hiiesalu *et al.* 2012). Therefore, the findings from simple pot or field experiments may not be neatly extended to natural grasslands. Assuming that priority effects exist, we predict that many subordinate plant species will share mycorrhizal associations with the most dominant perennial plant (i.e. *H. comata*). With many grass tussocks per metre square, *H. comata* was often associated with relatively divergent AMF communities compared with other plant species including an annual grass (*B. japonicus*) and biennial forb (*T. dubius*) (Fig. 1).

The observed variation in AMF communities among the eight most dominant grassland plants (Figs 1 and 2) is likely of ecological importance and may help explain the coexistence of dominant plant species. Grassland ecosystems are often dominated by several grass species (Sims and Risser 2000) which tend to share traits due to shared ancestry (e.g. Davies *et al.* 2013). Plants with similar traits are likely to have similar niches and may engage in intense resource competition which may lead to competitive exclusion (Burns and Strauss 2011; Violle *et al.* 2011; but see Bennett *et al.* 2013). As AMF effectively extend plant roots and increase resource acquisition, grasses that associate with distinct AMF may experience enhanced functional complementarity relative to other grasses. Functional complementarity may be partly possible because individual AMF species are known to have host-specific effects (e.g. Van Der Heijden *et al.* 1998*a*; Klironomos 2003). Associations with divergent AMF may enable the more complete utilization of available resources and an overall increase in plant productivity (Van Der Heijden *et al.* 1998*b*; Montesinos-Navarro *et al.* 2012*a*). Increased niche partitioning among plants may partially explain how grasslands can be so productive despite their low levels of phylogenetic diversity (Bennett *et al.* 2013). Other studies have shown that AMF may influence plant coexistence (reviewed by Hart *et al.* 2003). However, effects of AMF on plant coexistence can range from positive (Hart *et al.* 2003; Van Der Heijden *et al.* 2003) to negative (Hartnett *et al.* 1993), and to neutral (Eissenstat and Newman 1990).

## Conclusions

Across the three replicate grassland sites, we detected significant effects of both site and plant species on AMF communities. We found that related plant species (mostly grasses) tended to have more divergent AMF communities than distantly related plant species, suggesting that functional complementarity and local coexistence of otherwise ecologically similar grasses may be increased by association with distinct AMF taxa.

## Sources of Funding

This research was made possible in part through a grant to K.O.R. from the National Parks Ecological Research Fellowship Program, a partnership between the National Parks Ecological Research Fellowship Program, funded through a grant from the Andrew W. Mellon Foundation helping form a partnership between the National Park Service, the Ecological Society of America and the National Park Foundation.

## Contributions by the Authors

The authors shared in constructing the phylogenies, analysing the data and writing.

## Conflicts of Interest Statement

None declared.

## Supporting Information

The following supporting information is available in the online version of this article –

**Appendix S1.** Additional methods and results for a fossil calibrated phylogeny.

**Appendix S2.** Additional methods information on the root collection and T-RFLP.

**Figure S1.** Relative abundance of arbuscular mycorrhizal fungi OTUs.

**Table S1.** Information on the operational taxanomic unit (OTU) clusters in the ‘knowns’ library.

## Supplementary Material

Supplementary Data
