# Supplementary material for: More closely related plants have more distinct mycorrhizal communities
Source: AoB Plants. 2014 Sep 23;6:plu051. doi: 10.1093/aobpla/plu051 (PMC4172195; doi:10.1093/aobpla/plu051)

Supporting information figure

Figure S1 Heat maps showing the relative abundance (square size) of roots with various arbuscular mycorrhizal fungi operational taxonomic units (OTU) by plant species for sites 1 (A), 2 (B), and 3 (C). The size of the squares is positively correlated with the number of roots with a specific OTU. Plant species abbreviations are as follows: Arfr = *Artemisia frigida*, Bogr= *Bouteloua gracilis*, Brja = *Bromus japonicus*. Cafi= *Carex filifolia*, Heco= *Hesperostipa comata*, Koma= *Koeleria macrantha*, Pasm= *Pascopyrum smithii*, and Trdu= *Tragopogon dubius.*

Fig. S1


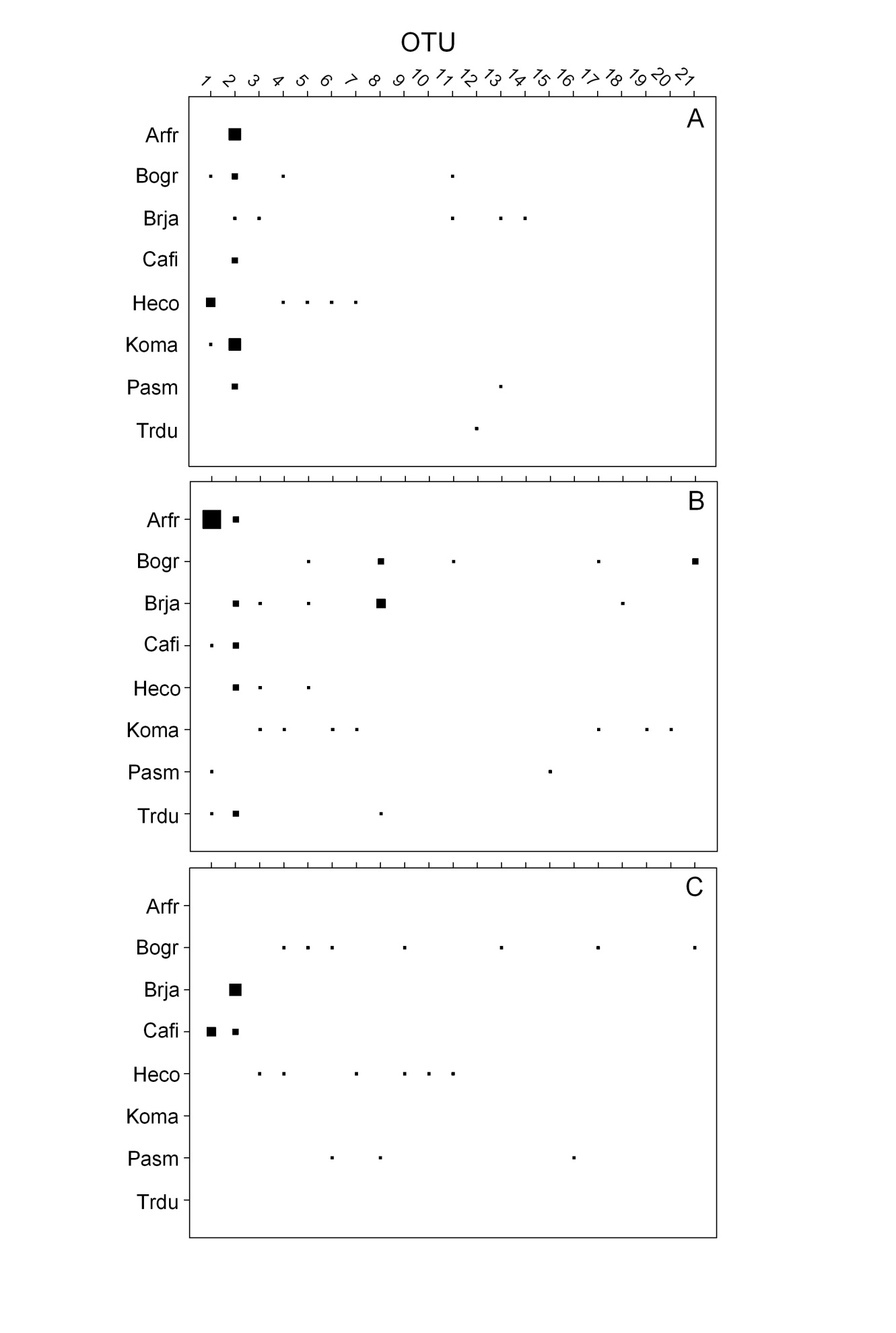

Supplement: Supplementary Data [file plu051_supplementary_data.zip › plu051supp_fig1.docx]
