# Supplementary material for: More closely related plants have more distinct mycorrhizal communities
Source: AoB Plants. 2014 Sep 23;6:plu051. doi: 10.1093/aobpla/plu051 (PMC4172195; doi:10.1093/aobpla/plu051)
Supplement: Supplementary Data [file plu051_supplementary_data.zip › plu051supp_table1.docx]

Supplemental information table.

Table S1 Information on the Organizational Taxanomic Unit (OTU) clusters in the “knowns” library/database identified from the 192 root samples.

| OTU | Description of OTU clusters | Source material | Reference |
| --- | --- | --- | --- |
| 1 | Unidentified arbuscular mycorrhizae, Custer County, Montana (MT) | 1,2 |  |
| 2 | Unidentified arbuscular mycorrhizae, Custer County, MT | 2 |  |
| 3 | Unidentified *Glomus*, North Dakota (ND) |  | (Jordan et al., 2012) |
| 4 | Unidentified *Glomus*, ND |  | (Jordan et al., 2012) |
| 5 | Unidentified *Glomus*, ND |  | (Jordan et al., 2012) |
| 6 | *Glomus intraradices*, *Glomus versiforme* |  | (Aldrich-Wolfe, 2007) |
| 7 | Unidentified *Glomus*, ND and Custer County, MT | 1 | (Jordan et al., 2012) |
| 8 | Unidentified *Glomus*, ND |  | (Jordan et al., 2012) |
| 9 | Unidentified *Glomus*, ND |  | (Jordan et al., 2012) |
| 10 | Unidentified *Glomus*, ND |  | (Jordan et al., 2012) |
| 11 | Unidentified *Glomus*, ND |  | (Jordan et al., 2012) |
| 12 | Unidentified *Glomus*, ND |  | (Jordan et al., 2012) |
| 13 | *Glomus caledonium*, *Glomus clarum*, *Glomus geosporum* |  | (Aldrich-Wolfe, 2007) |
| 14 | Unidentified *Glomus*, ND |  | (Jordan et al., 2012) |
| 15 | Unidentified *Glomus*, ND |  | (Jordan et al., 2012) |
| 16 | Unidentified *Glomus*, ND |  | (Jordan et al., 2012) |
| 17 | Unidentified arbuscular mycorrhizae, Custer County, MT | 1 |  |
| 18 | Unidentified *Glomus*, ND |  | (Jordan et al., 2012) |
| 19 | Unidentified arbuscular mycorrhizae, Custer County, MT | 1 |  |
| 20 | Unidentified *Glomus*, ND |  | (Jordan et al., 2012) |
| 21 | Unidentified *Glomus*, ND |  | (Jordan et al., 2012) |

1 Terminal restriction fragments from a single spore isolated from Custer County, Montana

2 Terminal restriction fragments from Sudan grass roots of potted plants maintaining a single species of arbuscular mycorrhizal fungi from Custer County, Montana

LITERATURE CITED

Aldrich-Wolfe L. 2007. Distinct mycorrhizal communities on new and established hosts in a transitional tropical plant community. *Ecology* 88: 559-566.

Jordan NR, Aldrich-Wolfe L, Huerd SC, Larson DL, Muehlbauer G. 2012. Soil-occupancy effects of invasive and native grassland plant species on composition and diversity of mycorrhizal associations. *Invasive Plant Science and Management* 5: 494-505.
