# Supplementary material for: More closely related plants have more distinct mycorrhizal communities
Source: AoB Plants. 2014 Sep 23;6:plu051. doi: 10.1093/aobpla/plu051 (PMC4172195; doi:10.1093/aobpla/plu051)
Supplement: Supplementary Data [file plu051_supplementary_data.zip › plu051supp_data2.docx]

Supporting information Appendix S2.

Additional methods information on the root collection and molecular identification of arbuscular mycorrhizal fungi and analyses.

*Root sampling*

There were apparent trade-offs in determining the optimal time to sample eight plant species with divergent phenologies (e.g. anthesis from May through September)—optimize the timing of sampling by a common developmental stage versus control for seasonal/abiotic driven changes in intraradical and extraradical hyphae. Others established that root colonization (e.g. Bentivenga & Hetrick 1992) and AMF associations (e.g. Helgason et al. 1999) vary seasonally. Here we timed collection of plant roots by phenology of individual plant species. We sampled plants during anthesis because preliminary trials indicated that AMF colonization declined as plants senesced. This provided an important control for ultimately comparing the AMF associated with roots of varying species and sites during a phase of active growth and demand for nutrients. Roots were sampled from May 6 through September 28, 2009 on randomly selected individuals at each of three sites. Plants were randomly collected from around one 0.1-ha plot per site, excavated from the soil to a depth of 15 cm, brought to the lab, soil was separated from the roots, and 10 fine root segments (1 cm length) were dried in a dessicator at room temperature.

*DNA extraction*

DNA was extracted from root tissue using Ultra Clean Plant DNA isolation kit (MoBio Laboratories, Inc., Carlsbad, California, USA). Changes to the manufacturer’s protocol were increased duration in the 65°C water bath (15 and not 10 minutes) and centrifuging of samples (1 minute not 30 seconds). DNA extracts were kept at -20°C in manufacturer’s storage buffer of solution P5 (50μl) for <2.5 years.

*PCR1*

PCR1 consisted of a total volume of 50.0 μL, containing 4.0 μL of SSU-Glom1 (2.5 μM) (Eurofins MWG Operon, Huntsville, Alabama, USA), 4.0 μL of LSU-Glom1 (2.5 μM) (Eurofins MWG Operon), 1 μL genomic DNA extract, and components of the Taq PCR Core Kit (Qiagen, Hilden, Germany). Specifically, we added 5.0 μL of 10x Coralload buffer, 1.0 μL of dNTPs (10mM), 10 μL Q Solution, and 0.25 μL of *Taq* polymerase. A positive control consisted of DNA extracted from a store bought mushroom (*Agaricus* sp.), and the negative control was sterilized water. PCR was performed on a PTC-100 hot bonnet thermocycler (MJ Research Inc., Waltham, MA, USA) for 35 cycles: 94° C for 2 minutes; 35 cycles of 92° C for 30 seconds, 55° C for 30 seconds, 72° C for 1 minute; and a final extension period at 72° C for 5 minutes. The *Alu*I restriction digest consisted of a total volume of 10.0 μL, containing,1 μL of 10x buffer, 0.1 μL of BSA (0.1%), 0.4 μL of *Alu*I (Promega, Madison, WI, USA), and 5 μL of PCR1 template.

This was incubated overnight at 37°C and covered to prevent light from degrading the primers.

*PCR2*

PCR2 portion used *Alu*I digested products which were then fluorescently labeled with the universal fungal primers 6-FAM-ITS4 and HEX-ITS5 (Eurofins MWG Operon) (White et al., 1990). PCR2 followed the same recipe and thermocycler settings as PCR1 with the labeled primers. The PCR2 amplicons were then purified using the Ultra Clean Plant DNA Isolation kit (MoBio Laboratories, Inc.). After DNA clean-up, the PCR2 amplicons were restriction digested with *Hinf*I (Promega) and *Mbo*I (New England Biolabs Inc., Ipswich, Massachusetts, USA). The *Hinf*I restriction digest consisted of a total volume of 10.0 μL, containing 1 μL of 10x buffer, 0.1 μL BSA (0.1%), 0.4 μL *Hinf*I, and 5 μL of PCR2 template. This was incubated overnight at 37°C and covered to prevent light from degrading the primers. The *Mbo*I restriction digest consisted of a total volume of 10.0 μL, containing 1 μL of 10x buffer, 0.2 μL *Mbo*I, and 5 μL of PCR2 template. This was incubated for 4 hrs at 37°C and covered to prevent light from degrading the primers.

Restriction digested amplicons were submitted along with undigested amplicons to the Roy J. Carver Biotechnology Center (University of Illinois, Urbana, Illinois, USA) for T-RFLP analysis using Applied Biosystems 3730xl Genetic Analyzer (Applied Biosystems, Foster City, California, USA). ROX1000 was used as the size standard.

*Knowns database*

AMF in root samples from the field were identified by comparing lengths of terminal restriction fragments (TRFs) and PCR2 amplicons with lengths obtained from multiple sources for known AMF species and unknown AMF samples. The database consisted of TRF and PCR2 amplicon profiles of known AMF and some unknown AMF. Each profile comprised four data points: the length of PCR2 amplicon (5’), the *Hinf*I-digested TRFs (5’ and 3’), and the *Mbo*I-digested TRFs (3’) (sensu Aldrich-Wolfe, 2007). Similar to another study, we combined local reference samples with a broad database of samples (Aldrich-Wolfe, 2007). Local reference samples consisted of PCR2 amplicon and TRFs of individual mycorrhizal spores from Sudan grass trap cultures or roots of Sudan grass culturing single AMF species of unknown identity. Lengths were determined from nine AMF isolates from unknown AMF species growing in pure culture and 21 spores of unknown species produced from trap cultures of plant/soil samples from Custer County, Montana (see Table S1 in Supporting Information). The nested PCR protocol for spores followed that for roots, except that DNA was extracted by crushing the spore in the PCR tube immediately prior to PCR1. These data were combined with other lists of TRF and PCR2 amplicon lengths for known AMF from other studies (Aldrich-Wolfe, 2007; Jordan et al., 2012) to form a reference library for known and unknown AMF.

*Analysis*

Database T-RFLP is a useful approach to characterize AMF communities in part because taxa have relatively consistent lengths of PCR2 amplicons and terminal restriction fragments (TRFs) (Lekberg et al., 2007). The program TRAMPR implemented in the R environment was used to match PCR2 amplicons and TRFs from root samples with a database of knowns and to provide output for additional community analyzes. TRAMPR was used to cluster the knowns database/library (FitzJohn and Dickie, 2007). Peaks less than 50bp were removed. To standardize the data, we then removed peaks <10% of the largest peak (i.e. relative threshold of 10%). In other words, if the maximum peak was 10,000 relative fluorescence units (rfu) then we would retain all peaks greater than 1,000 rfus. To account for the range of fluorescence intensities observed across samples, a ratio of 1:10 appeared optimal for balancing the tradeoff between 1) need to remove sequencer noise and avoid false detection of AMF and 2) not removing actual TRF peaks indicative of AMF. An OTU was considered present if all four lengths (one PCR2 amplicon and three TRFs) were present within 1.5 bp of the known’s lengths.

Literature Cited

Aldrich-Wolfe L. 2007. Distinct mycorrhizal communities on new and established hosts in a transitional tropical plant community. *Ecology* 88: 559-566.

FitzJohn R, Dickie IA. 2007. TRAMPR: an R package for analysis and matching of terminal-restriction fragment length polymorphism (TRFLP) profiles. *Molecular Ecology Notes* 7: 583-587.

Jordan NR, Aldrich-Wolfe L, Huerd SC, Larson DL, Muehlbauer G. 2012. Soil-occupancy effects of invasive and native grassland plant species on composition and diversity of mycorrhizal associations. *Invasive Plant Science and Management* 5: 494-505.

Lekberg Y, Koide RT, Rohr JR, Aldrich-Wolfe L, Morton JB. 2007. Role of niche restrictions and dispersal in the composition of arbuscular mycorrhizal fungal communities. *Journal of Ecology* 95: 95-105.

White TJ, Bruns T, Lee S, Taylor J. 1990. Amplification and direct sequencing of fungal ribosomal RNA genes for phylogenetics. In: *PCR protocols: A guide to methods and applications*. Innis MA, Gelfand DH, Sninsky JJ, White TJ eds. San Diego, California, USA: Academic Press, 315-322.
