# Supplementary material for: More closely related plants have more distinct mycorrhizal communities
Source: AoB Plants. 2014 Sep 23;6:plu051. doi: 10.1093/aobpla/plu051 (PMC4172195; doi:10.1093/aobpla/plu051)
Supplement: Supplementary Data [file plu051_supplementary_data.zip › plu051supp_data1.docx]

Supporting information Appendix S1.

Additional methods and results for a fossil calibrated phylogeny.

Methods

*Plant phylogeny construction*

*Phylogenetic signal tests*

We separately tested whether plant phenology and AMF associations varied by plant phylogeny and exhibited a phylogenetic signal. To assess the degree phylogeny explains the phenological similarity of species (i.e. trait conservatism), we quantified phylogenetic signal using the descriptive *K* statistic (Blomberg et al., 2003) using the package Picante (Kembel et al., 2010) in R. Blomberg’s *K* statistic compares the observed signal for a plant trait (i.e. mean Julian day of anthesis across sites) to the signal under a Brownian motion process and provides an estimate of phylogenetic signal or trait conservatism (Blomberg et al., 2003). K values less than one indicate that species resemble each other less than expected under Brownian motion evolution; K values greater than one indicate that species resemble each other more than expected. Picante uses a permutation test to test the significance of the observed K value. To do this, the names of taxa are iteratively shuffled across the tips of the phylogeny 999 times. The significance of the *K* statistic is assessed with the quantile of the observed phylogenetically independent contrast variance versus the null distribution, which provides a 1-tailed P-value testing whether the phylogenetic signal is greater than expected. With only eight species, the significance test should be interpreted cautiously since tests with so few species are likely to fail to detect significant differences when they actually exist (type II errors)(Rezende et al., 2007) and justified use of a relaxed alpha level= 0.10.

To test for a phylogenetic signal in AMF host associations, we needed to determine whether variation in AMF distance (=community dissimilarity) among pairs of host plant species was positively related to the phylogenetic (ultrametric) distance between this pair of plant hosts. Phylogenetic distance between each pair of plant species was determined with the program Patristic (Fourment and Gibbs, 2006). AMF distance among pairs of plant species per site was measured using Jaccard distance implemented with the package vegan (Oksanen et al., 2010) in R. Prior to Jaccard distance calculations, we summed the sample data (i.e. binomial presence/absence data) by species per site. This produced quantitative data of AMF OTU associations per plant species per site. We treated sites as a form of landscape-level replication and averaged dissimilarity measures for each pair of species (e.g. *Artemisia frigida* and *Carex filifolia*) which reduced the data to 28 species pairs (8 plant spp. × [{8-1}/2]= 28). This approach ensures that our pairwise description of pairwise AMF distance is not site-specific. We used linear regression (LR) to determine the direction of the relationship between AMF distance and phylogenetic distance. A Mantel test was used to account for the non-independence in the data (not accounted for with LR), caused by a given plant species being present in multiple pairwise species combinations (8 plant spp. but 28 pairwise comparisons) (e.g. Weiblen et al., 2006; Violle et al., 2011).

Results

*Phylogenetic signal tests*

Among the eight plant species, related species tended to have similar phenologies (i.e. anthesis)(phylogenetic signal test, *K*= 0.51, P= 0.095) suggesting trait conservatism. The phylogenetic distance of pairs of plants, however, was negatively correlated with AMF distance (R_2_= 0.13, F_1,26_= 3.89, P= 0.059) which was significant according to a Mantel test (Pearson's product-moment correlation, r= -0.36, P= 0.014). The Mantel test is the more robust analysis since it accounts for the non-independence in the data. In conclusion, related pairs of plants tended to have divergent AMF.

Literature Cited

Blomberg SP, Garland T, Jr., Ives AR. 2003. Testing for phylogenetic signal in comparative data: behavioral traits are more labile. *Evolution* 57: 717-745.

Fourment M, Gibbs MJ. 2006. PATRISTIC: a program for calculating patristic distances and graphically comparing the components of genetic change. *BMC Evolutionary Biology* 6: 1-5.

Kembel SW, Cowan PD, Helmus MR, Cornwell WK, Morlon H, Ackerly DD, Blomberg SP, Webb CO. 2010. Picante: R tools for integrating phylogenies and ecology. *Bioinformatics* 26: 1463-1464.

Oksanen J, Blanchet FG, Kindt R, Legendre P, O'Hara RB, Simpson GL, Solymos P, Stevens MHH, Wagner H. 2010. Vegan: Community Ecology Package.

Rezende EL, Lavabre JE, Guimarães PR, Jordano P, Bascompte J. 2007. Non-random coextinctions in phylogenetically structured mutualistic networks. *Nature* 448: 925-928.

Violle C, Nemergut DR, Pu Z, Jiang L. 2011. Phylogenetic limiting similarity and competitive exclusion. *Ecology Letters* 14: 782-787.

Weiblen GD, Webb CO, Novotny V, Basset Y, Miller SE. 2006. Phylogenetic dispersion of host use in a tropical insect herbivore community. *Ecology* 87: S62-S75.
